# Validation of a primary care electronic medical records case definition for eczema: retrospective cross-sectional study

**DOI:** 10.1186/s13223-023-00785-4

**Published:** 2023-05-28

**Authors:** Hannah Stirton, Leanne Kosowan, Elissa M Abrams, Jennifer LP Protudjer, John Queenan, Alexander Singer

**Affiliations:** 1 Division of Dermatology, Department of Medicine, University of Toronto, Toronto, ON Canada; 2 Department of Family Medicine, Rady Faculty of Health Sciences, University of Manitoba, Winnipeg, Manitoba Canada; 3 Department of Pediatrics, Section of Allergy and Clinical Immunology, Rady Faculty of Health Sciences, University of Manitoba, Winnipeg, Manitoba Canada; 4 Department of Pediatrics, Division of Allergy and Immunology, University of British Columbia, Vancouver, BC Canada; 5 Department of Pediatrics and Child Health, Rady Faculty of Health Sciences, University of Manitoba, Winnipeg, MB Canada; 6 Institute of Environmental Medicine, Karolinska Institute, Stockholm, Sweden; 7 Department of Family Medicine, Queens University, Kingston, Ontario Canada

**Keywords:** Eczema, Primary Health Care, Electronic Health Records

## Abstract

**Background:**

To validate case definitions for eczema using primary care Electronic Medical Record (EMR) data from the Canadian Primary Care Sentential Surveillance Network (CPCSSN).

**Methods:**

This study used EMR data from 1,574 primary care providers in seven Canadian provinces, representing 689,301 patients. Using a subset of patient records seven medical students or family medicine residents created a reference set of 1,772 patients. A total of 23 clinician-informed case definitions were validated against the reference. We assessed agreement using sensitivity (SE), specificity (SP), positive predictive value (PPV), negative predictive value (NPV) and overall accuracy. The case definitions with the best agreement statistics were deployed to estimate the prevalence of eczema in the CPCSSN.

**Results:**

Case definition 1 had the highest SE (92.1%,85.0-96.5) but a lower SP (88.5%,86.7–90.1) and PPV (36.6%,33.1–40.3). Case definition 7 was the most specific case definition with a SP (99.8%, 99.4–100) and PPV (84.2%,61.2–94.7) but low SE (15.8%,9.3–24.5). Case definition 17 had a SE (75.3%, 65.7–83.3), SP (93.8%, 91.5–94.3) and PPV 43.7% (38.3–49.2). When we applied the most specific and most sensitive case definitions, we estimate the prevalence of eczema to be between 0.8 and 15.1%. Case definition 17 suggests an eczema prevalence estimate of 8.2% (8.08–8.21%).

**Conclusions:**

We validated EMR-based eczema case definitions to estimate the prevalence of clinician-documented eczema. Future studies may choose to apply one or more of these definitions’ dependent on their studies objectives to inform disease surveillance as well as explore burden of illness or interventions related to eczema care in Canada.

## Background

Eczema (atopic dermatitis) is a common inflammatory skin disease characterized by intense pruritus, xerosis and recurrent eczematous lesions [1]. The prevalence of eczema is increasing globally making it the most burdensome skin disorder worldwide [2–6]. The pathophysiology of eczema involves complex interactions between genetics and the immune system interacting with environmental and infectious agents [7]. Eczema is the initial step in the “atopic triad” (i.e. a person with eczema, asthma and allergic rhinitis). Having eczema increases the risk of having other atopic conditions such as asthma and allergic rhinitis [7]. There is also a known association between eczema and food allergy [7] as well as several non-atopic conditions including depression, anxiety, and attention deficit hyperactivity disorder (ADHD) [5, 6, 8]. Risk factors for developing eczema include a family history of atopy, female sex, Black race, and high socioeconomic status [1, 7, 9–14]. A worldwide study found that eczema affects approximately 5–20% of children [15]. While traditionally thought of as a childhood disease, recent evidence suggests eczema in adults is common [13, 16, 17].

There are several features of eczema that make its epidemiology challenging to study, such as non-standardized nomenclature, variable morphology and heterogenous skin lesion distribution [12]. Thus far, most research on eczema has largely relied on survey data. Due to the complexity and diversity of this disease the true prevalence of eczema may be over- or underestimated. Application of a validated case definition to large representative datasets can provide primary care provider diagnosed prevalence estimates that can increase our current understanding of risk factors and comorbidities of eczema. Several prior studies have attempted to use Hanifin’s and Rajakin’s criteria and/or the UK Working Group criteria for diagnosing atopic dermatitis [18–21]. Previous literature [18–21] has focused on specific ICD-9 codes within a specific cohort of patients. Building on this work we test and validate possible definitions of eczema using both specific and less specific diagnostic coding applied to data derived from 11 different Canadian primary care EMRs. We aimed to include definitions that would range from being highly specific but potentially less sensitive, to highly sensitive and less specific.

## Methods

### Study design

We conducted a retrospective cross-sectional study to develop and validate an EMR-based case definition for eczema. Using EMR medical record review we created a reference set for validation. Case definitions were applied within a pan-Canadian representative patient population [22]. We used the checklist of reporting criteria for validation studies [23].

### Setting

This study used de-identified EMR data from 1,574 primary care providers participating in the Canadian Primary Care Sentinel Surveillance Network (CPCSSN). These family physicians, nurse practitioners and community pediatricians are located across seven Canadian provinces, British Columbia, Alberta, Manitoba, Ontario, Quebec, Nova Scotia, and Newfoundland and Labrador. There were 11 EMR vendors represented in this data extract.

### Data sources

The CPCSSN generates a pan-Canadian repository using EMR data extracted and processed from each provincial network. CPCSSN captures longitudinal primary care EMR data representing > 1,800,000 Canadians. Data are included for all patients that attend an appointment with a consenting provider. Patients do have the option to opt-out of CPCSSN upon request. The data in the CPCSSN repository is processed using computerized coding and cleaning algorithms [24–26]. During the data cleaning process, invalid entries are deleted, and the data are standardized to map prescribed medications to Anatomical Therapeutic Chemical (ATC) Classification codes, laboratory variable names to Logical Observation Identifiers Names and Codes (LOINC) codes, and medical diagnoses to International Classification of Disease, ninth edition, clinical modification (ICD-9-CM) codes. The repository includes both structured data fields as well as short-text fields with diagnoses, medications, allergies, and risk factors. Regionally some provincial networks hold free-text encounter notes. A de-identification process is applied to all free text to render the data anonymized. This study accessed the billing, health condition (problem list), encounter diagnosis, medication, allergy, patient, and provider tables from the CPCSSN repository as well as free-text data from the Manitoba regional network.

### Participants

We utilized EMR records for active patients, defined as those with at least one appointment between January 1, 2017, and December 31, 2019 [27]. There were 689,301 active patients in CPCSSN with health records from inception of the EMR to December 31, 2019.

### Reference dataset

**Fig. 1:**
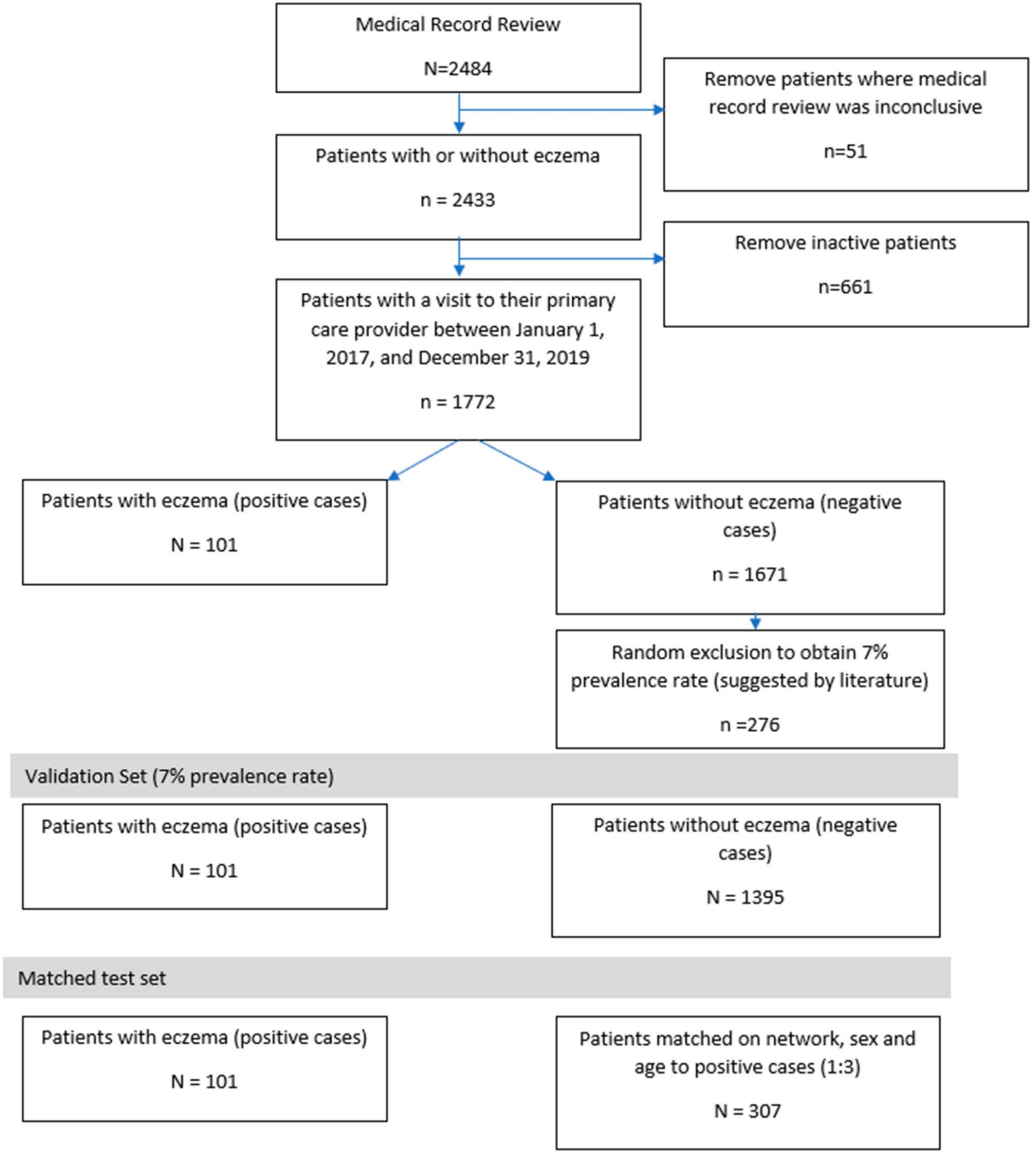
Flow diagram for creation of the eczema reference set from the Canadian Primary Care Research Network (CPCSSN)

We created a sub-set of CPCSSN patient records for medical records review. Medical record review was performed by seven medical students and family medicine residents. Medical students/residents reviewed encounter notes and clinician free-text entries in the health condition, billing, encounter diagnosis tables for patients with an ICD-9-CM code 691 (atopic dermatitis and related conditions), or 692 (contact dermatitis and other eczema) in the EMR (n = 358,560 encounter notes, 2,292 free-text entries) to create a positive reference set. Medical students/residents reviewed an additional 27,630 randomly selected records to create a negative reference set. This created a reference set of 2,484 patients (Fig. 1). The reference standard used for algorithm development assigned each patient as positive, negative or unsure for a diagnosis of eczema [21]. Two students/residents reviewed each set of records, and discrepancies between the students/residents were reviewed by a family physician or allergist. There were 51 patients excluded due to an inability to differentiate between eczema and other rashes using eczema diagnostic criteria [21], and another 661 patients removed because they did not have an appointment in the previous two years [27]. Our final reference set had 1,772 patients (101 positive, and 1,671 negative) (Fig. 1). Studies from the US have suggested a prevalence rate of 7% [12, 13, 16] we therefore randomly excluded 275 patients producing our validation set of 1,496 (101 positive, 1,395 negative). We included data on each patient including province, age, and sex. Patient age was calculated at the index date of December 31, 2019. To create a test data set we matched positive and negative cases at a 1:3 ratio using province, sex and age creating a dataset of 408 patients (101 positive, 307 negative) (Fig. 1).

### Case definitions

Case definitions were developed by clinicians and researchers to include ICD-9-CM and ATC codes found in the health condition, billing, encounter diagnoses and medication tables (Table 1). Case definitions were informed by diagnostic criteria [21], and previous case validation studies [18–20]. ICD-9-CM 691.8 is specific for eczema. However, some providers may use the code 692.9 (contact dermatitis and other eczema, unspecific cause) [18] or less specific codes (691 (atopic dermatitis and related conditions), 692 (contact dermatitis and other eczema)) with or without sub-codes. Diagnostic criteria and previous studies suggest an association with atopic conditions and allergy [18–21]. We assessed the health conditions, billing and encounter diagnosis tables for indications of asthma (ICD-9-CM 493) [25], or rhinitis/hay fever (ICD-9-CM 472, 477) [26]. Within the EMR, primary care providers can indicate with short-text if the patient has an allergy. We applied a previously validated algorithm to identify patients with documentation of an allergy in the EMR [26, 27]. Allergy documentation in the EMR may reference a specific allergy such as a drug, food, stinging insect, vaccine, environment, or other allergy [26, 27]. We assessed prescriptions for topical eczema medications (ATC: D07, D02A, D11AH) [28].

**Table 1:** EMR-based Case Definitions for Identification of Eczema

Case definition	Description
Case definition 1	≥ 1 health condition, billing or encounter diagnosis for ICD-9-CM code starting with 691.xx or 692.xx
Case definition 1b	≥ 1 health condition, billing or encounter diagnosis for 3-digit ICD-9-CM code 691 or 692
Case definition 2	≥ 1 health condition for 3-digit ICD-9-CM code 691, 692 OR ≥ 2 billing/Encounter Diagnosis for 3-digit ICD-9-CM code 691, 692
Case definition 3	≥ 1 health condition, billing or encounter diagnosis for ICD-9-CM code starting with 691.xx or 692.xx OR ≥ 1 medication for ATC code starting with D07, D02A, D11AH
Case definition 4	≥ 1 health condition for ICD-9-CM code starting with 691.xx, 692.xx OR ≥ 2 billing encounter diagnosis for ICD-9-CM starting with 691.xx, 692.xx OR ≥ 1 medication for ATC code starting with D07, D02A, D11AH
Case definition 5	≥ 1 health condition, billing or encounter diagnosis for 3-digit ICD-9-CM 691
Case definition 6	≥ 1 health condition, billing or encounter diagnosis for 3-digit ICD-9-CM 692
Case definition 7	≥ 1 health condition, billing or encounter diagnosis for ICD-9-CM 691.8
Case definition 8	≥ 1 health condition, billing or encounter diagnosis for ICD-9-CM 691.8 OR ≥ 1 health condition, billing or encounter diagnosis for 3-digit ICD-9-CM 691, 692
Case definition 9	≥ 1 health condition, billing or encounter diagnosis for ICD-9-CM 691.8 OR ≥ 1 health condition, billing or encounter diagnosis for 3-digit ICD-9-CM 691, 692 AND ≥ 1 medication for ATC code starting with D07, D02A, D11AH
Case definition 10	≥ 1 health condition, billing or encounter diagnosis for ICD-9-CM 691.8 OR ≥ 1 medication for ATC code starting with D07, D02A, D11AH
Case definition 11	≥ 1 health condition, billing or encounter diagnosis for ICD-9-CM 691.8 or 692.9 OR ≥ 1 health condition, billing or encounter diagnosis for 3-digit ICD-9-CM 691, 692 AND ≥ 1 medication for ATC code starting with D07, D02A, D11AH
Case definition 12	≥ 1 health condition, billing or encounter diagnosis for ICD-9-CM 691.8 or 692.9 OR ≥ 1 medication for ATC code starting with D07, D02A, D11AH
Case definition 13	≥ 1 health condition, billing or encounter diagnosis for ICD-9-CM 691.8 or 692.9
Case definition 14	≥ 1 health condition, billing or encounter diagnosis for ICD-9-CM 691.8 or 692.9 OR ≥ 1 health condition, billing or encounter diagnosis for 3-digit ICD-9-CM 691 or 692
Case definition 15	≥ 1 health condition, billing or encounter diagnosis for ICD-9-CM code starting with 691.xx or 692.xx AND ≥ 1 health condition, billing or encounter diagnosis for atopy condition ICD-9-CM starting with 493.xx (asthma) or 472.xx/477.xx (rhinitis/hay fever) OR ≥ 1 allergy documented in the allergy table of the EMR
Case definition 16	≥ 1 health condition, billing or encounter diagnosis for ICD-9-CM code starting with 691.xx or 692.xx AND ≥ 1 health condition, billing or encounter diagnosis for atopy condition ICD-9-CM starting with 493.xx (asthma) or 472.xx/477.xx (rhinitis/hay fever) OR ≥ 1 food allergy documented in the allergy table of the EMR
Case definition 17	≥ 1 health condition for 3-digit ICD-9-CM code 691 or 692 OR ≥ 2 billing/Encounter Diagnosis for 3-digit ICD-9-CM code 691 or 692 OR ≥ 1 health condition, billing or encounter diagnosis for ICD-9-CM 691.8 or 692.9
Case definition 18	≥ 1 health condition, billing or encounter diagnosis for ICD-9-CM 691.8 or 692.9 OR ≥ 1 health condition, billing or encounter diagnosis for ICD-9-CM code starting with 691.xx or 692.xx AND ≥ 1 health condition, billing or encounter diagnosis for atopy condition ICD-9-CM starting with 493.xx (asthma) or 472.xx/477.xx (rhinitis/hay fever) OR ≥ 1 health condition, billing or encounter diagnosis for ICD-9-CM code starting with 691.xx or 692.xx AND ≥ 1 allergy documented in the allergy table of the EMR
Case definition 19	≥ 1 health condition, billing or encounter diagnosis for 3-digit ICD-9-CM code 691 or 692 AND ≥ 1 health condition, billing or encounter diagnosis for atopy condition ICD-9-CM starting with 493.xx (asthma), or 472.xx/477.xx (rhinitis/hay fever) OR ≥ 1 allergy documented in the allergy table of the EMR
Case definition 20	≥ 1 health condition, billing or encounter diagnosis for 3-digit ICD-9-CM code 691 or 692 AND ≥ 1 health condition, billing or encounter diagnosis for atopy condition ICD-9-CM starting with 493.xx (asthma), or 472.xx/477.xx (rhinitis/hay fever) OR ≥ 1 food allergy documented in the allergy table of the EMR
Case definition 21	≥ 1 health condition, billing or encounter diagnosis for ICD-9-CM 691.8 or 692.9 OR ≥ 1 health condition, billing or encounter diagnosis for 3-digit ICD-9-CM code 691 or 692 AND ≥ 1 health condition, billing or encounter diagnosis for atopy condition ICD-9-CM starting with 493.xx (asthma), or 472.xx/477.xx (rhinitis/hay fever) OR ≥ 1 allergy documented in the allergy table of the EMR
Case definition 22	≥ 1 health condition, billing or encounter diagnosis for 3-digit ICD-9-CM code 691 or 692 AND ≥ 1 health condition, billing or encounter diagnosis for atopy condition ICD-9-CM starting with 493.xx (asthma), or 472.xx/477.xx (rhinitis/hay fever) OR ≥ 1 allergy documented in the allergy table of the EMR OR ≥ 1 medication for ATC code starting with D07, D02A, D11AH
Case definition 23	≥ 1 health condition, billing or encounter diagnosis for ICD-9-CM 691.8 or 692.9 OR ≥ 1 health condition, billing or encounter diagnosis for 3-digit ICD-9-CM code 691 or 692 AND ≥ 1 health condition, billing or encounter diagnosis for atopy condition ICD-9-CM starting with 493.xx (asthma), or 472.xx/477.xx (rhinitis/hay fever) OR ≥ 1 health condition, billing or encounter diagnosis for 3-digit ICD-9-CM code 691 or 692 AND ≥ 1 allergy documented in the allergy table of the EMR OR ≥ 1 health condition, billing or encounter diagnosis for 3-digit ICD-9-CM code 691 or 692 AND ≥ 1 medication for ATC code starting with D07, D02A, D11AH

### Statistical analysis

We assessed agreement between each of the case definitions and the two reference sets (test and validation) with several metrics including sensitivity, specificity, positive predictive value (PPV), negative predictive value (NPV), and overall accuracy. The equations for these metrics are presented below.

$$\begin{array}{*{20}{c}}{PPV}&{\frac{{TP}}{{TP + FP}}}\end{array}$$

$$\begin{array}{*{20}{c}}{Sensitivity}&{\frac{{TP}}{{TP + FN}}}\end{array}$$

$$\begin{array}{*{20}{c}}{NPV}&{\frac{{TN}}{{TN + FN}}}\end{array}$$

$$\begin{array}{*{20}{c}}{Specificity}&{\frac{{TN}}{{TN + FP}}}\end{array}$$

$$\begin{array}{*{20}{c}}{Accuracy}&{\frac{{TP + TN}}{{TP + FP + FN + TN}}}\end{array}$$

*TP: true positive, FP: false positive, FN: false negative, FP: false positive*

The prevalence and 95% confidence limits were computed using an exact binomial test. We assessed associations between case definition capture and patient characteristics including age, sex, atopic comorbidities and medication using chi-square, and t-test. Significance was assessed at 0.05. Statistical analyses were conducted using SAS V9.4 (SAS Institute Inc, Cary, NC).

## Results

The final reference set included positive and negative cases from the following Canadian provinces: British Columbia, Alberta, Manitoba, Ontario, Quebec. In the test set there were 101 positive and 307 negative patients matched using province, sex and age. Positive and negative cases were not significantly different based on, urban or rural location (0.6287), or annual visit frequency (0.8366). We assessed the agreement between our reference set and twenty-three case definitions (Table 1). Eczema-specific ICD-9-CM codes 691.8 and 692.9 had low sensitivity and high specificity. Case definition 7 (ICD-9-CM 691.8) had a sensitivity 15.8% and specificity 100% (Table 2). ICD-9-CM 691.8 or 692.9 (case definition 13) had a sensitivity 59.4%, and specificity 94.1%. Inclusion of related but less specific codes demonstrated an overall improvement in capture. Case definition 1 included all related ICD-9-CM codes with a sensitivity 92.1%, specificity 91.9%, PPV 78.8% and NPV 97.2%. Case definitions 14 and 17 saw improvements in specificity (93.8% and 93.8%) and PPV (82.1% and 80.0%) compared to case definition 1 (Table 2). Incorporating medications that can be used to treat eczema and related atopic conditions did not improve agreement.

**Table 2:** Validation of Eczema Case Definitions in the Test Dataset n = 408

Case Definition	True Positive	True Negative	False Negative	False Positive	SE	SP	PPV	NPV	ACC
1	93	282	8	25	92.1 (85.0, 96.5)	91.9 (88.2, 94.7)	78.8 (71.8, 84.5)	97.2 (94.8, 98.6)	91.9 (88.8, 94.4)
1b	28	305	73	2	27.7 (19.3, 37.5)	99.4 (97.7, 99.9)	93.3 (77.3, 98.3)	80.7 (78.7, 82.5)	81.6 (77.5, 85.3)
2	16	305	85	2	15.8 (9.3, 24.5)	99.4 (97.7, 99.9)	88.9 (65.2, 97.2)	78.2 (76.7, 79.6)	78.7 (74.4, 82.6)
3	50	269	51	38	49.5 (39.4, 59.6)	87.6 (83.4, 91.1)	56.8 (47.9, 65.3)	84.1 (81.2, 86.5)	78.2 (73.9, 82.1)
4	42	269	59	38	41.6 (31.9, 51.8)	87.6 (83.4, 91.1)	52.5 (43.1, 61.7)	82.0 (79.4, 84.4)	76.2 (71.8, 80.3)
5	19	306	82	1	18.8 (11.7, 27.8)	99.7 (98.2, 100)	95.0 (72.0, 99.3)	78.9 (77.3, 80.4)	79.7 (75.4, 83.5)
6	14	306	87	1	13.9 (7.8, 22.2)	99.7 (98.2, 100)	93.3 (65.1, 99.1)	77.9 (76.5, 79.2)	78.4 (74.1, 82.3)
7	16	307	85	0	15.8 (9.3, 24.5)	100 (98.8, 100)	100	78.3 (76.9, 79.7)	79.2 (74.9, 83.0)
8	44	305	57	2	43.6 (33.7, 53.8)	99.4 (97.7, 99.9)	95.7 (84.5, 98.9)	84.3 (81.8, 86.4)	85.5 (81.8, 88.8)
9	25	305	76	2	24.8 (16.7, 34.3)	99.4 (97.7, 99.9)	24.8 (20.6, 29.2)	80.1 (78.2, 81.8)	80.9 (76.7, 84.6)
10	43	269	58	38	42.6 (32.8, 52.8)	87.6 (83.4, 91.1)	53.1 (43.8, 62.2)	82.3 (79.6, 84.7)	76.5 (72.1, 80.5)
11	69	288	32	19	68.3 (58.3, 77.2)	93.8 (90.5, 96.2)	78.4 (69.7, 85.1)	90.0 (87.1, 92.3)	87.5 (83.9, 90.6)
12	67	257	34	50	66.3 (56.3, 75.4)	83.7 (79.1, 87.7)	57.3 (50.1, 64.2)	88.3 (85.1, 90.9)	79.4 (75.2, 83.2)
13	60	289	41	18	59.4 (49.2, 69.1)	94.1 (90.9, 96.5)	76.9 (67.4, 84.3)	87.6 (84.8, 89.9)	85.5 (81.8, 88.8)
14	87	288	14	19	86.1 (77.8, 92.2)	93.8 (90.5, 96.2)	82.1 (74.6, 87.7)	95.4 (92.7, 97.1)	91.9 (88.8, 94.4)
15	27	301	74	6	26.7 (18.4, 36.5)	98.1 (95.8, 99.3)	81.8 (65.7, 91.4)	80.3 (78.3, 82.1)	80.4 (76.2, 84.1)
16	23	301	78	6	22.8 (15.0, 32.2)	98.1 (95.8, 99.3)	79.3 (61.6, 90.2)	79.4 (77.6, 81.1)	79.4 (75.2, 83.2)
17	76	288	25	19	75.3 (65.7, 83.3)	93.8 (90.5, 96.2)	80.0 (71.8, 86.3)	92.0 (89.1, 94.2)	89.2 (85.8, 92.1)
18	70	286	31	21	69.3 (59.3, 78.1)	93.2 (89.7, 95.7)	76.9 (68.4, 83.7)	90.2 (87.3, 92.5)	87.3 (83.6, 90.3)
19	11	306	90	1	10.9 (5.6, 18.7)	99.7 (98.2, 100)	91.7 (59.0, 98.8)	77.3 (76.1, 78.5)	77.7 (73.3, 81.6)
20	9	306	92	1	8.9 (4.2, 16.2)	99.7 (98.2, 100)	90.0 (53.6, 98.6)	76.9 (75.8, 78.0)	77.2 (72.8, 81.2)
21	70	288	31	19	69.3 (59.3, 78.1)	93.8 (90.5, 96.2)	78.7 (70.1, 85.3)	90.3 (87.4, 92.6)	87.8 (84.2, 90.8)
22	44	305	57	2	43.6 (33.7, 53.8)	99.4 (97.7, 99.9)	95.7 (84.5, 98.9)	84.3 (81.8, 86.4)	85.5 (81.8, 88.8)
23	77	288	24	19	76.2 (66.7, 84.1)	93.8 (90.5, 96.2)	80.2 (72.1, 86.4)	92.3 (89.4, 94.5)	89.5 (86.1, 92.3)

**Table 3:** Validation of Eczema Case Definitions in the Validation Dataset n = 1495

Case Definition	True Positive	True Negative	False Negative	False Positive	SE	SP	PPV	NPV	ACC
1	93	1234	8	161	92.1 (85.0, 96.5)	88.5 (86.7, 90.1)	36.6 (33.1, 40.3)	99.4 (98.8, 99.7)	88.7 (87.0, 90.3)
1b	28	1328	73	67	27.7 (19.3, 37.5)	95.2 (93.9, 96.3)	29.5 (22.0, 38.2)	94.8 (94.2, 95.4)	90.6 (89.1, 92.1)
2	16	1379	85	16	15.8 (9.3, 24.5)	98.9 (98.1, 99.3)	50.0 (34.0, 66.0)	94.2 (93.7, 94.6)	93.3 (91.9, 94.5)
3	50	1165	51	230	49.5 (39.4, 59.6)	83.5 (81.5, 85.4)	17.9 (14.7, 21.5)	95.8 (95.0, 96.5)	81.2 (79.1, 83.2)
4	42	1190	59	205	41.6 (31.9, 51.8)	85.3 (83.3, 87.1)	17.0 (13.6, 21.1)	95.3 (94.5, 96.0)	82.4 (80.3, 84.3)
5	19	1361	82	34	18.8 (11.7, 27.8)	97.6 (96.6, 98.3)	35.9 (24.9, 48.6)	94.3 (93.8, 94.8)	92.3 (90.8, 93.6)
6	14	1359	87	36	13.9 (7.8, 22.2)	97.4 (96.5, 98.2)	28.0 (17.8, 41.1)	94.0 (93.5, 94.4)	91.8 (90.3, 93.1)
7	16	1392	85	3	15.8 (9.3, 24.5)	99.8 (99.4, 100)	84.2 (61.2, 94.7)	94.3 (93.8, 94.7)	94.1 (92.8, 95.3)
8	44	1325	57	70	43.6 (33.7, 53.8)	95.1 (93.8, 96.1)	38.6 (31.4, 46.4)	96.0 (95.2, 96.6)	91.7 (90.2, 93.0)
9	25	1360	76	35	24.8 (16.7, 34.3)	97.5 (96.5, 98.3)	41.7 (30.8, 53.4)	94.7 (94.1, 95.2)	92.6 (91.1, 93.9)
10	43	1197	58	198	42.6 (32.8, 52.8)	85.8 (83.9, 87.6)	17.8 (14.3, 22.0)	95.4 (94.6, 96.1)	82.9 (80.9, 84.8)
11	69	1282	32	113	68.3 (58.3, 77.2)	91.9 (90.3, 93.3)	37.9 (32.9, 43.2)	97.6 (96.8, 98.2)	90.3 (88.7, 91.8)
12	67	1147	34	248	66.3 (56.3, 75.4)	82.2 (80.1, 84.2)	21.3 (18.4, 24.4)	97.1 (96.2, 97.8)	81.2 (79.1, 83.1)
13	60	1310	41	85	59.4 (49.2, 69.1)	93.9 (92.5, 95.1)	41.4 (35.2, 47.8)	97.0 (96.2, 97.6)	91.6 (90.1, 92.9)
14	87	1249	14	146	86.1 (77.8, 92.2)	89.5 (87.8, 91.1)	37.3 (33.4, 41.5)	99.0 (98.2, 99.3)	89.3 (87.6, 90.8)
15	27	1347	74	48	26.7 (18.4, 36.5)	96.6 (95.5, 97.5)	36.0 (26.9, 46.3)	94.8 (94.2, 95.4)	91.8 (90.3, 93.2)
16	23	1355	78	40	22.8 (15.0, 32.2)	97.1 (96.1, 97.9)	36.5 (26.4, 48.0)	94.6 (94.0, 95.1)	92.1 (90.6, 93.4)
17	76	1297	25	98	75.3 (65.7, 83.3)	93.0 (91.5, 94.3)	43.7 (38.3, 49.2)	98.1 (97.4, 98.7)	91.8 (90.3, 93.1)
18	70	1283	31	112	69.3 (59.3, 78.1)	92.0 (90.4, 93.3)	38.5 (33.4, 43.8)	97.6 (96.9, 98.2)	90.4 (88.8, 91.9)
19	11	1368	90	27	10.9 (5.6, 18.7)	98.1 (97.2, 98.7)	29.0 (17.2, 44.4)	93.8 (93.4, 94.2)	92.2 (90.7, 93.5)
20	9	1374	92	21	8.9 (4.2, 16.2)	98.5 (97.7, 99.1)	30.0 (16.8, 47.7)	93.7 (93.4, 94.1)	92.5 (91.0, 93.7)
21	70	1285	31	110	69.3 (59.3, 78.1)	92.1 (90.6, 93.5)	38.9 (33.8, 44.3)	97.6 (96.9, 98.2)	90.6 (89.0, 92.0)
22	44	1325	57	70	43.6 (33.7, 53.8)	95.0 (93.7, 96.1)	38.6 (31.4, 46.4)	95.9 (95.1, 96.5)	91.5 (90.0, 92.9)
23	77	1263	24	132	76.2 (66.7, 84.1)	90.5 (88.9, 92.0)	36.8 (32.4, 41.5)	98.1 (97.4, 98.7)	89.6 (87.9, 91.1)

In the validation set there were 101 positive and 1395 negative patients representing an eczema prevalence of 6.8%. Positive cases were significantly more likely to be female compared to male (0.0076). However, urban vs. rural residency (0.4427), age (0.89) or annual visit frequency (0.365) were not significantly different. Case definition 7 (ICD-9-CM 691.8) had a strong specificity 99.8% and PPV 84.2% but low sensitivity 15.8%. All other case definitions had a low PPV including case definition 13 (ICD-9-CM 691.8, 692.9) with a PPV of 41.4%. Case definition 1 had a sensitivity 92.1%, specificity 88.5%, PPV 36.6%, NPV 99.4% and accuracy 88.7%. Case definition 14 and 17 demonstrated slight decreases in sensitivity (86.1%, 75.3%) but increases in specificity (89.5%, 93.0%), PPV (37.3%, 43.7%), and accuracy (89.3%, 91.8%) (Table 3). Incorporating medications that can be used to treat eczema and related atopic conditions did not improve our case definitions.

Within the CPCSSN dataset of 689,301 active patients, the estimated prevalence of eczema ranged from 0.8% (0.79–0.83%) in our most specific definition to 15.1% (15.05–15.22%) in our most liberal definition (Table 4). Case definition 17 estimates a lifetime prevalence of 8.2% (8.08–8.21%). Inclusion of atopic conditions and medications (case definition 23) suggested a prevalence of 9.8% (9.71–9.85%). Application of the case definitions with the strongest metrics suggests patients captured were significantly more likely to be female, prescribed a medication that can treat eczema and have asthma or rhinitis (Table 4). Interestingly, patients with eczema in case definition 7, 13 or 17 were less likely to have an allergy documented in the EMR.

**Table 4:** Application of the Strongest Eczema Case Definitions to the CPCSSN Repository N = 689,301 patients

Case definition	Prevalence n(%, 95%CI)	Female patient, n(%)	Patient age, mean(SD)	Eczema medication n (%)	Patients with an allergy n (%)	Patients with asthma n(%)	Patients with rhinitis n(%)
Case definition 1	104,354 (15.1%, 15.05–15.22%)	**65,085 (62.4%)**	**53.9 (19.9)**	**27,885 (26.7%)**	14,948 (14.3%)	**18,973 (18.2%)**	**11,151 (10.7%)**
Case definition 7	5569 (0.8%, 0.79–0.83%)	**3479 (62.5%)**	**48.9 (20.3)**	**1620 (29.1%)**	**447 (8.0%)**	**1228 (22.1%)**	**786 (14.1%)**
Case definition 13	28,762 (4.2%, 4.13–4.22%)	**18,240 (63.4%)**	**51.9 (20.0)**	**8452 (29.4%)**	**2892 (10.1%)**	**5426 (18.9%)**	**4208 (14.6%)**
Case definition 14	97,980 (14.2%, 14.13–14.3%)	**61,258 (62.5%)**	**53.9 (19.9)**	**26,301 (26.8%)**	14,260 (14.6%)	**17,998 (18.4%)**	**10,429 (10.6%)**
Case definition 17	56,171 (8.2%, 8.08–8.21%)	**35,434 (63.1%)**	**53.4 (20.1)**	**15,275 (27.2%)**	**7504 (13.4%)**	**11,007 (19.6%)**	**7167 (12.8%)**
Case definition 23	67,416 (9.8%, 9.71–9.85%)	**42,467 (63.0%)**	**52.3 (20.0)**	**26,301 (39.0%)** [1]	**14,260 (21.2%)** ^1^	**17,998 (26.7%)** ^1^	**10,429 (15.5%)** ^1^
Patients not captured in an eczema case definition	584,538 (84.8%)	322,481 (55.2%)	52.0 (19.3)	55,506 (9.5%)	84,878 (14.5%)	64,811 (11.1%)	31,863 (5.5%)

^1^medication, allergy, asthma and rhinitis diagnosis are included in the case definition

Boldface font indicated the variable was significant at p-value > 0.05

95%CI: 95% confidence intervals

## Discussion

We validated case definitions for eczema in the CPCSSN repository of primary care patients. Using the EMR-based case definitions suggests a prevalence of eczema between 0.8 and 15.1% comparable to prior study estimates [12, 13, 16, 29]. Multiple US surveys suggest the adult prevalence of eczema to be 7.0% [12, 13, 16, 30, 31]. In our study case definition 17 suggests a slightly higher lifetime prevalence of 8.2%.

Overall, the case definitions performed well in the test set of matched patients with case definitions 1, 11, 14, 17 and 23 demonstrating metrics over 70%. As anticipated the specificity (100%) and PPV (100%) was high in case definition 7 (eczema specific ICD-9-CM 691.8) but the sensitivity was low (15.8%). When we included both ICD-9-CM 691.8 or 692.9 (case definition 13) the specificity and PPV dropped slightly (94.1% and 76.9%, respectively) with a moderate increase in sensitivity (59.4%). Similar to Hsu and colleagues, we found that patients with 692.9 have eczema [18].

When we tested our case definitions in the validation set the PPV of each definition decreased. The most specific definition (case definition 7) had a specificity 99.8% and PPV 84.2%, with a sensitivity of 15.8%. The use of ICD-9-CM 691.8 alone was able to identify patients who have eczema according to our reference set. However, as suggested by Hsu and colleagues the use of 691.8 is overly restrictive and underestimates the prevalence of eczema [18]. Previously studies using specific ICD-9-CM codes also reported low sensitivity [18, 19] and/or PPV [18]. Low sensitivity from 4-digit ICD-9-CM codes (691.8 and 692.9) likely resulted from frequent use of less specific 3-digit ICD-9-CM codes. Reliance on 4-digit codes may exclude billing data from provincial systems that only require 3-digit codes. Case definitions 14 and 17 used eczema-specific as well as 3-digit ICD-9-CM codes.

When we compared overall metrics, case definition 17, despite low PPV (43.7%) was able to maintain higher specificity (93.0%) and sensitivity (75.3%). Although the sensitivity was lower then reported by case definition 1 (92.1%) and 14 (86.1%) overall case definition 17 found substantially less false positives. Contrary to Hsu and colleagues we found that requiring multiple occurrences of less specific ICD-9-CM codes improved performance [18]. The addition of related atopic conditions and medications to our case definition did not substantially change sensitivity (76.2%), specificity (90.5%), or PPV (36.8%).

Depending on a study’s objectives, researchers may require application of one or more of the above-described case definitions. For example, a clinical trial would require a definition with high specificity, whereas for epidemiological studies definitions with a more balanced sensitivity and specificity and high accuracy would be preferred. Case definition 1 or 17 may be useful for epidemiology and surveillance efforts. Unconfirmed diagnoses could be related to a lack of formal criteria as well as provider uncertainty in making the official diagnosis for the purpose of billing [18]. Case definition 1 and 17 will include some false positives and is likely inclusive of some patients with ill-defined eczematous disorders or non-atopic dermatosis conditions. However, the estimated prevalence of case definition 17 (8.2%), is similar to previous literature [12, 13, 16]. Reliance on diagnostic information are much simpler and more transferable than those requiring medications or atopic diagnoses. Additionally, simpler definitions may be more desirable given known variations in data quality [44, 45] and provider and system factors such as EMR capabilities or provincial billing code requirements.

The validity and utility of the case definitions can be further supported by the characteristics of the patients captured. Independent of the case definition applied we found evidence that patients captured by one of these case definitions are experiencing or had experienced some form of atopic or non-atopic dermatoid condition. In all case definitions there was significant increases in diagnosis of other atopic conditions including asthma, and rhinitis consistent with other literature [1, 19, 26, 32−43]. Although patients with eczema did not have higher rates of food allergy, a previous study using CPSCNN data also noted lower then expected allergy prevalence suggesting incomplete documentation of allergy in primary care EMRs [26]. While we cannot discern if medications are prescribed for the treatment of eczema specifically, patients meeting criteria received more eczema related medications.

### Limitations

One of the key limitations is that we relied on primary care provider documentation in EMR, which could both overestimate or underestimate eczema prevalence due to variation in provider coding, missing diagnoses, and incomplete documentation. Clinicians use EMR systems for clinical purposes and may not be concerned with the use of specific ICD-9-CM codes for secondary purposes. For example, the ICD-9-CM code 692 includes the term ‘eczema,’ but also contains multiple branch points that refer to contact dermatitis from a variety of sources including detergents, chemicals, drugs and medicines, among others. Furthermore, within typical medical practice, eczema may refer to atopic dermatitis or non-atopic dermatoses. CPCSSN includes only primary care EMR data and does not represent specialist visits. Future studies linking this dataset to representative cohorts of allergist and dermatologist could be helpful to improve the certainty and accuracy of our prevalence estimates.

## Conclusions

Application of a validated EMR-based case definition for eczema can improve health surveillance of this increasingly prevalent condition. Future research should explore the burden of illness, trends and interventions related to eczema care using these validated case definitions.

## Data Availability

Datasets generated and/or analysed during the current study are not publicly available due to the confidential nature of data governed by the PHIA legislation but are available from the corresponding author on reasonable request and with the appropriate approvals.
